# Comparative Metabolomics Analysis of Four Pineapple (*Ananas comosus* L. Merr) Varieties with Different Fruit Quality

**DOI:** 10.3390/plants14152400

**Published:** 2025-08-03

**Authors:** Ping Zheng, Jiahao Wu, Denglin Li, Shiyu Xie, Xinkai Cai, Qiang Xiao, Jing Wang, Qinglong Yao, Shengzhen Chen, Ruoyu Liu, Yuqin Liang, Yangmei Zhang, Biao Deng, Yuan Qin, Xiaomei Wang

**Affiliations:** 1 Fujian Provincial Key Laboratory of Haixia Applied Plant Systems Biology, Haixia Institute of Science andTechnology, College of Life Sciences, Fujian Agriculture and Forestry University, Fuzhou 350002, China; zhengping13@mails.ucas.ac.cn (P.Z.); jiahao.mu@outlook.com (J.W.); lidenglin016@163.com (D.L.); xieshiyu927628@163.com (S.X.); caixinkai2003@163.com (X.C.); qiangxiao_five@163.com (Q.X.); wangjing051402@163.com (J.W.); yql_0418@163.com (Q.Y.); chenshengzhen654@163.com (S.C.); 2 Horticulture Research Institute, Guangxi Academy of Agricultural Sciences, Nanning Investigation Station of South Subtropical Fruit Trees, Ministry of Agriculture, Nanning 530004, China; dengbiao3333@163.com; 3 Pingtan Science and Technology Research Institute, College of Marine Sciences, Fujian Agriculture and Forestry University, Fuzhou 350002, China; liuruoyu2013@foxmail.com; 4 Xiamen Botanical Garden, Xiamen 361000, China; liangyuqin5@sina.com; 5 Yunnan Institute of Tropical Crops, Jinghong City, Xishuangbanna 666100, China; yangmeiz66@163.com

**Keywords:** pineapple, metabolome, fruit quality, comparative analysis

## Abstract

Understanding the metabolic characteristics of pineapple varieties is crucial for market expansion and diversity. This study performed comparative metabolomic analysis on the “Comte de Paris” (BL) and three Taiwan-introduced varieties: “Tainong No. 11” (XS), “Tainong No. 23” (MG), and “Tainong No. 13” (DM). A total of 551 metabolites were identified across the four varieties, with 231 metabolites exhibiting no significant differences between all varieties. This included major sugars such as sucrose, glucose, and fructose, as well as key acids like citric, malic, and quinic acids, indicating that the in-season maturing fruits of different pineapple varieties can all achieve good sugar–acid accumulation under suitable conditions. The differentially accumulated metabolites (DAMs) that were identified among the four varieties all primarily belonged to several major subclasses, including phenolic acids, flavonoids, amino acids and derivatives, and alkaloids, but the preferentially accumulated metabolites in each variety varied greatly. Specifically, branched-chain amino acids (L-leucine, L-isoleucine, and L-valine) and many DAMs in the flavonoid, phenolic acid, lignan, and coumarin categories were most abundant in MG, which might contribute to its distinct and enriched flavor and nutritional value. XS, meanwhile, exhibited a notable accumulation of aromatic amino acids (L-phenylalanine, L-tryptophan), various phenolic acids, and many lignans and coumarins, which may be related to its unique flavor profile. In DM, the dominant accumulation of jasmonic acid might contribute to its greater adaptability to low temperatures during autumn and winter, allowing off-season fruits to maintain good quality. The main cultivar BL exhibited the highest accumulation of L-ascorbic acid and many relatively abundant flavonoids, making it a good choice for antioxidant benefits. These findings offer valuable insights for promoting different varieties and advancing metabolome-based pineapple improvement programs.

## 1. Introduction

Pineapple (*Ananas comosus* L. Merr.) is the most important economic crop in the Bromeliaceae family, ranking as the world’s third-largest tropical fruit [1]. Pineapple is cultivated in over 90 countries and regions, covering more than 400,000 hectares, with an annual trade volume of more than 2.5 billion US dollars [2]. Pineapple fruit is valued for its rich nutritional content, strong aroma, juicy and tender flesh, and balanced sweet-sour taste, making it highly popular [3]. In China, which ranks fourth globally in terms of cultivation area, pineapple cultivation has a long history. The main cultivated varieties include “Comte de Paris”, smooth Cayenne, “Shenwan”, etc. However, with increasing consumer demand for fruit diversity and high quality, challenges including limited variety options, genetic degradation, poor fruit quality, short storage periods, and low economic returns greatly impede the sustainable development of the pineapple industry [2]. The breeding and/or introduction of new varieties has become a key measure in recent years to enhance the market competitiveness of mainland varieties. China’s Taiwan region has made notable advancements in pineapple hybrid breeding, leading to the development of the “Tainong” series of varieties, characterized by their diversity, superior genetic traits, high quality, and substantial economic value [2]. In recent years, many pineapple varieties such as “Tainong No. 11”, “Tainong No. 13” “Tainong No. 16”, and “Tainong No. 23” have been introduced on the mainland, and the cultivation areas of these promoted varieties have been increasing [4,5,6]. A systematic assessment of the quality characteristics of different varieties is a necessary step before the industrial promotion of these newly introduced varieties.

Fruit quality is a crucial factor influencing sales, and evaluating fruit quality based on physicochemical parameters alone is not enough for a comprehensive understanding. With the development of systematic biology, metabolomics techniques are widely applied in studies related to the comprehensive evaluation of fruit quality and its formation mechanism. Comparative metabolomics has been widely applied to reveal metabolic differences between different varieties in guava (*Psidium guajava* L.) fruit [7], longan (*Dimocarpus longan* Lour.) pulp [8], coffee (*Coffea arabica* L.) [9], and citrus [10]. Moreover, metabolomics techniques have also been applied in the investigation of pineapple, including identifying the characteristic metabolites for each cultivar [11], monitoring ripening-specific metabolites [12,13], the formation of the sweet-acidic taste during fruit development [14], dynamic alterations in fruit color and quality [15], water accumulation-mediated flesh translucency [16], postharvest internal browning [17], the flower induction mechanism [18], potentially bioactive metabolites from pineapple waste extracts [19], D-leaf signatures of different varieties [20], and low temperature stress response [21]. The composition of metabolites is closely linked to fruit quality, and distinct variations in metabolite composition and fruit quality exist among different varieties [7,9]. While metabolomics has been extensively employed in investigations related to pineapple fruit quality, further metabolomic analyses of newly introduced superior pineapple varieties are still necessary to provide crucial guidance for their widespread application.

The pineapple industry significantly contributes to the economies of tropical and subtropical regions in China, necessitating a growing demand for diverse pineapple varieties [2]. To expand the pineapple market and attract consumers to a diversity of pineapple types, data on the nutritional potential of different pineapple varieties are needed. In the present study, comparative metabolic analysis was conducted on four representative pineapple varieties, including “Comte de Paris”, the dominant cultivated variety in China, and three promoted varieties introduced from Taiwan, China. We discuss the specific metabolic profiling of each pineapple variety and highlight their nutritional and health potentials. These findings will serve as a foundation for the promotion of each variety and provide useful information for metabolome-assisted breeding and plant biology in pineapple.

## 2. Results

### 2.1. Fruit Phenotyping of the Four Representative Pineapple Varieties

**Figure 1:**
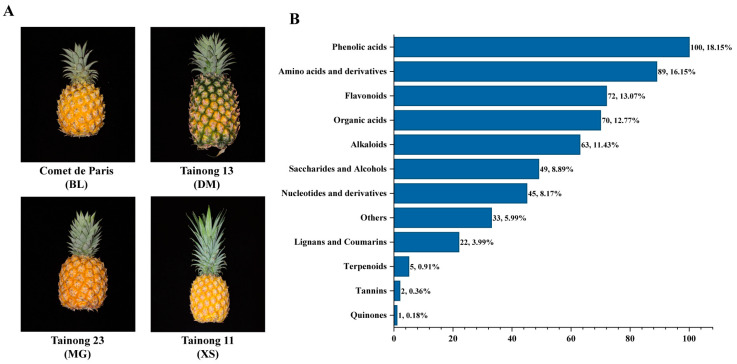
Pineapple fruit phenotyping and metabolite profiling. (**A**) Fruit of the four representative pineapple varieties; (**B**) Classification of all identified metabolites.

In this study, three promoted pineapple varieties introduced from Taiwan, China were selected for comparative metabolomic analysis, with the dominant cultivated variety “Comte de Paris” (BL) used as a control (Figure 1A, Table 1). Among these varieties, (1) “Comte de Paris” (BL) was the main cultivated pineapple variety in China, bred from the Queen group lineage. It exhibited vigorous growth and strong adaptability to climatic stress. The mature fruit had yellow to deep yellow flesh with a sweet, fragrant taste and a crisp texture with minimal fiber. The soluble solid content was 17.8 ± 1.8%, and the titratable acid content was 0.61 ± 0.07%; (2) “Tainong 23” (MG), a hybrid of “Tainong 19” and “Tainong 21,” produced fruit with golden flesh and a distinctive coconut and mango flavor. The flesh was fine-grained, sweet, and juicy, with an average soluble solid content of 17.9% and an average titratable acidity of 0.56%; (3) “Tainong 11” (XS), a hybrid of “Smooth Cayenne” and “Yellow Mauritius”, was renowned for its uniquely delightful aroma and was also referred to as the “perfume pineapple.” The flesh was fine-grained and low in fiber, with a soluble solid content of 17.2 ± 1.8%. It had a slightly acidic taste, with an organic acid content of 0.60 ± 0.07%; and (4) “Tainong 13” (DM), which was characterized by sweetness, juiciness, and low acidity, with an average soluble solid content of 18.1 ± 1.2% and an average titratable acidity of 0.53 ± 0.12%. Compared to other varieties, this cultivar was more suitable for autumn and winter production and maintained excellent fruit quality even during the off-season.

### 2.2. Metabolic Profiling Analysis of Four Pineapple Varieties

**Figure 2:**
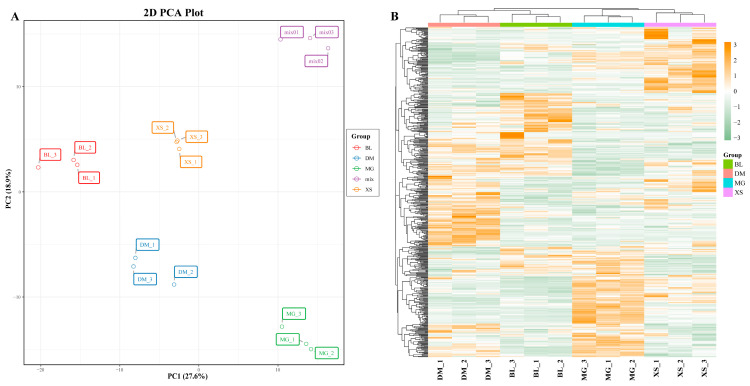
Principal component analysis (PCA) and hierarchical clustering heatmap of all pineapple samples. (**A**) PCA score plot of all pineapple samples with three quality control (mix 01–03); (**B**) Total metabolite clustering heatmap of all samples. Each column is a different sample, and each row is an identified metabolite. The heatmap was created based on the relative content value of identified metabolites and normalized by row. Differences in metabolite accumulation changes are shown in color as the scale, red for high abundance and green for low abundance.

Metabolite detection was performed on 12 fruit pulp samples representing four pineapple varieties using UPLC-MS/MS. A total of 551 metabolites were identified, including 100 phenolic acids, 89 amino acids and derivatives, 72 flavonoids, 70 organic acids, 63 alkaloids, 49 saccharides and alcohols, 45 nucleotides and derivatives, 22 lignans and coumarins, five terpenes, two tannins, and one quinone, and 33 other metabolites (Figure 1B, Table S1). Principal component analysis (PCA) was employed to gain initial insight into the overall metabolic differences among varieties and the degree of variability between replicates of the same variety. Figure 2A displays the score plot of the two principal components, PC1 and PC2, extracted from the PCA, where mix 01–03 represents the three quality control samples. PC1 and PC2 explained 27.6% and 18.9% of the total variance in the dataset, respectively. The three biological replicates for each pineapple variety formed close clusters, suggesting little variation between samples within each variety and indicating the high repeatability and reliability of our study. A notable separation was observed between samples from different pineapple varieties, signifying substantial metabolic differences among them. The hierarchical clustering heatmap further highlighted evident phenotypic variations in the accumulation of metabolites between the four pineapple varieties (Figure 2B). Collectively, the distinct metabolite compositions indicated that the four pineapple varieties exhibited unique metabolic profiles.

### 2.3. Identification of Differentially Accumulated Metabolites (DAMs)

**Figure 3:**
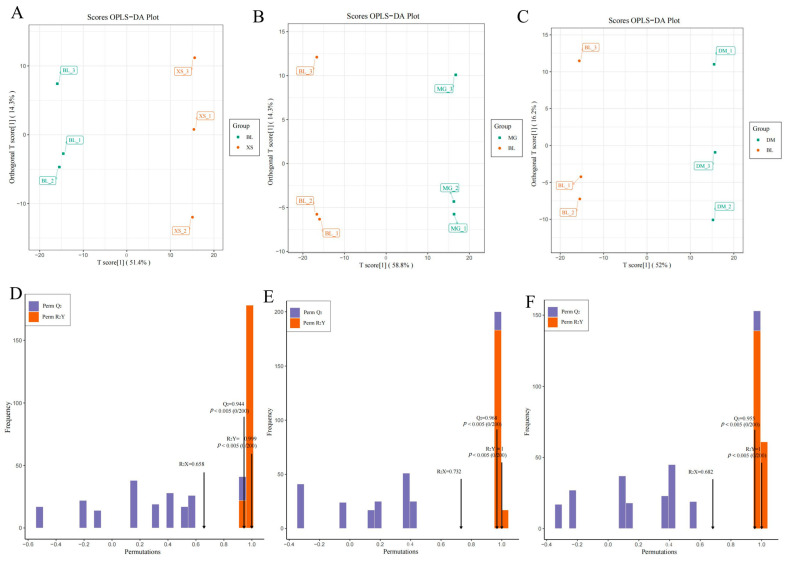
OPLS-DA analyses of three comparison groups with BL pineapple. (**A**–**C**) OPLS-DA score plots for BL vs. DM (**A**), BL vs. MG (**B**), and BL vs. XS (**C**), respectively. The abscissa represents the predicted principal component, and the ordinate represents the orthogonal principal components. The percentage indicates how well the component explains the data set. (**D**–**F**) Permutation test of the OPLS-DA model for BL vs. DM (**D**), BL vs. MG (**E**), and BL vs. XS (**F**), respectively. The abscissa represents the accuracy of the model, and the ordinate is the frequency of the model classification effect.

To assess the potential roles of metabolites in defining specific characteristics within the designated pineapple varieties, we initially employed the OPLS-DA model for pairwise comparisons. This approach could maximize the differentiation between varieties, facilitating the identification of differential metabolites. Score plots for six comparison groups were generated based on the metabolomic data analyzed using the OPLS-DA model (Figure 3 and Figure S1). The results showed the R^2^Y and Q^2^ values surpassed 0.92 in all comparison groups, indicating the OPLS-DA models had robust predictive capability and suitability for further screening of differential metabolites.

To further identify metabolites associated with fruit quality differences, differentially accumulated metabolites (DAMs) were identified through pairwise comparisons between the four pineapple varieties (six comparison groups in total), with each comparison conducted between two varieties only. Metabolites with VIP values ≥ 1.0 from the OPLS-DA model, and log2FC ≥1 (more-accumulated) or ≤−1.0 (less-accumulated), were defined as significant DAMs between certain comparative groups. A total of 231 metabolites showed no significant differences across all six comparative groups (Table S1). The results indicated that more than half of the metabolites in the saccharides and alcohols category (34, 69.4%), including glucose, fructose, sucrose, lactulose, melibiose, turanose, and panose, did not exhibit significant differences in pairwise comparisons across all four varieties. As to the DAMs in this category, most of them were more highly accumulated in the MG variety, including D-fucose, 1, 10-decanediol, D-sorbitol, mannitol, and dulcitol. Similarly, the majority of organic acids (48, 68.6%), including key acids in pineapple such as citric acid, isocitric acid, quinic acid, trans-citridic acid, cis-aconitic acid, L-(-)-malic acid, fumaric acid, succinic acid *, and pyruvic acid, showed no significant differences in pairwise comparisons among the varieties. Among the DAMs in this category, growth regulation related jasmonic acid was most abundant in DM, followed by BL, XS, and MG, while 1-naphthylacetic acid was most abundant in MG, followed by XS, DM, and BL. Furthermore, over half of the nucleotides and derivatives (45, 51.1%) also showed no significant variation across all six comparison groups. On the other hand, the majority of metabolites in the amino acids and derivatives category (54 out of 89) showed significant differences in comparisons among the different varieties. Specifically, 21 out of the 54 DAMs in this category were most abundant in the XS variety, including L-glutamine, L-lysine, L-arginine, L-phenylalanine, and L-tryptophan. While 18 out of the 54 DAMs in this category were most abundant in the MG variety, such as L-valine, L-leucine, L-isoleucine, L-proline and L-homocysteine. Furthermore, 13 out of the 54 DAMs in this category were most abundant in the DM variety, including L-phenylalanyl-L-phenylalanine, L-alanine, and S-adenosyl-L-methionine, whereas only two DAMs in this category were most abundant in the BL variety. The composition of secondary metabolites was also crucial for fruit quality formation [22,23,24]. We found that the majority of metabolites in various secondary metabolism categories, including flavonoids, phenolic acids, alkaloids, lignans, and coumarins, exhibited significant changes across different comparative groups (Table S1). Furthermore, 271 out of the 551 metabolites were annotated in the KEGG database, while the DAMs identified between different varieties were significantly enriched in metabolic pathways, such as phenylpropanoid biosynthesis, isoflavonoid biosynthesis, folate biosynthesis, valine, leucine, and isoleucine degradation, isoquinoline alkaloid biosynthesis, lysine degradation, and tryptophan metabolism (Table S2). The metabolite accumulation levels in these pathways may be closely associated with differences in fruit quality among pineapple varieties, and may require further exploration.

### 2.4. Differences in the Metabolic Profiles of the Three Introduced Varieties Compared to BL

**Figure 4:**
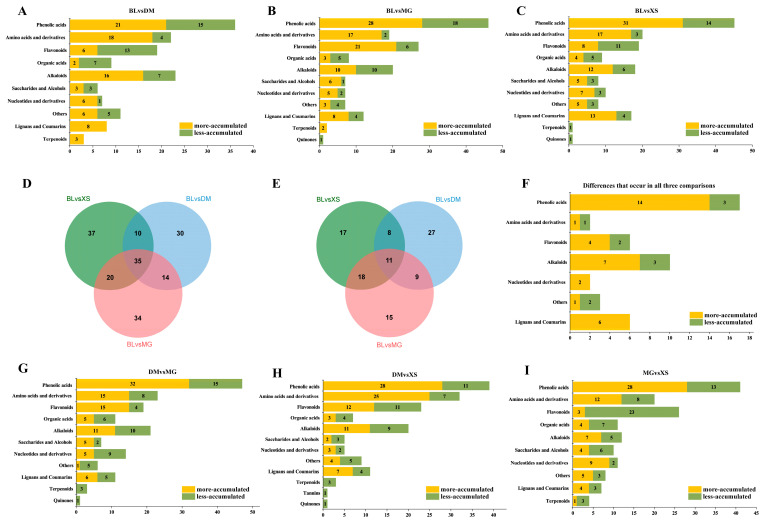
DAMs identified in different categories between four varieties. (**A**–**C**) Numbers of DAMs in different categories between BL vs. DM (**A**), BL vs. MG (**B**), and BL vs. XS (**C**), respectively. (**D**) Venn diagram of the common more-accumulated metabolites of BL vs. DM, BL vs. MG, and BL vs. XS. (**E**) Venn diagram of less-accumulated metabolites of BL vs. DM, BL vs. MG, and BL vs. XS. (**F**) Number of the common DAMs in different categories between BL vs. DM, BL vs. MG, and BL vs. XS. (**G**–**I**) Numbers of DAMs in different categories between DM vs. MG (**G**), DM vs. XS (**H**), and MG vs. XS (**I**), respectively.

To explore the metabolic differences between three introduced varieties and the main cultivated variety BL, differential metabolites were identified using BL as the control. Specifically, 144 significantly different metabolites were found between BL and DM (DM had 89 more-accumulated and 55 less-accumulated DAMs), including 36 phenolic acids, 23 alkaloids, 22 amino acids and derivatives, and 19 flavonoids (Figure 4A). Additionally, there were 156 significantly different metabolites between BL and MG (MG had 103 more-accumulated and 53 less-accumulated DAMs), encompassing 46 phenolic acids, 27 flavonoids, 20 alkaloids, and 19 amino acids and derivatives (Figure 4B). Furthermore, 156 significantly different metabolites were observed between BL and XS (XS had 102 more-accumulated and 54 less-accumulated DAMs), including 45 phenolic acids, 20 amino acids and derivatives, 19 flavonoids, 18 alkaloids, and 17 lignans and coumarins (Figure 4C). In comparison with the main variety BL, the number of more-accumulated DAMs in the three introduced varieties consistently exceeded the number of less-accumulated DAMs. These identified DAMs primarily belonged to several categories with higher metabolic diversity, such as phenolic acids, amino acids and derivatives, flavonoids, and alkaloids. In addition, many DAMs were also distributed in the lignans and coumarins category, which has a relatively low number of metabolites (only 22 in total). When sorted by relative abundance values in different varieties, metabolites in the lignans and coumarins category were found to be either most abundant in XS or most abundant in MG (Table S1). Particularly in the BL_XS comparison group, many DAMs originated from the lignans and coumarins category (Figure 4C). In addition, 35 common more-accumulated metabolites were identified in the three comparison groups, including 34 phenolic acids, seven alkaloids, six lignans and coumarins, four flavonoids, two nucleotides and derivatives, one amino acid and derivative, and one other metabolite (Figure 4D,F, Table S1). These metabolites mainly included antioxidants such as scopoletin, ferulic acid, and epigallocatechin-3-gallate, as well as compounds related to fruit flavor, such as 2-phenylethanol. Meanwhile, 11 common less-accumulated metabolites were identified in the three comparison groups, including three phenolic acids, three alkaloids, two flavonoids, one amino acid and derivatives, and two other metabolites. These consistently less-accumulated metabolites included L-ascorbic acid (vitamin C), p-coumaraldehyde, and sinapic acid (Figure 4E,F, Table S1).

### 2.5. Differences in the Metabolic Profiles Between the Three Introduced Varieties

To explore the differences among the introduced varieties, we also conducted pairwise comparisons of the metabolic composition of the three introduced varieties. The results showed that there were 163 significantly differential metabolites between DM and MG (MG had 95 more-accumulated and 68 less-accumulated DAMs), including 47 phenolic acids, 23 amino acids and derivatives, 21 alkaloids, and 19 flavonoids (Figure 4G). Between DM and XS, there were 156 significantly differential metabolites (XS had 95 more-accumulated and 61 less-accumulated DAMs), including 39 phenolic acids, 32 amino acids and derivatives, 23 flavonoids, and 20 alkaloids (Figure 4H). Moreover, 150 significantly differential metabolites were found between XS and MG (MG had 73 more-accumulated and 77 less-accumulated DAMs), including 41 phenolic acids, 26 flavonoids, 20 amino acids and derivatives, and 12 alkaloids (Figure 4I). Similarly, the DAMs in different comparison groups mainly belong to a few large categories, including phenolic acids, flavonoids, amino acids and derivatives, and alkaloids, which are closely related with their high diversity of metabolites within these categories. However, the comparison results among different varieties still exhibit significant differences. For instance, in the comparison between XS and MG, most of the 26 DAMs in the flavonoids category were more abundant in MG (23), with only three being more abundant in XS. On the other hand, among the 41 DAMs in the phenolic acids category, the majority were more abundant in XS (28), and only 13 were more abundant in MG. In the comparison between DM and MG, most of the DAMs in the phenolic acids and flavonoids categories were more abundant in MG. In the comparison between DM and XS, most DAMs in the phenolic acids and amino acids and derivatives categories were more abundant in XS. These results demonstrate that the dominant accumulated categories and numbers of metabolites are distinctly different among different varieties, correlating with their unique fruit quality.

### 2.6. K-Means Analysis of Variety-Specific Metabolite Profiling Detection

K-means clustering analysis was employed to identify subclusters of metabolites with similar accumulation trends between different pineapple varieties. A total of 320 DAMs were classified into nine subclasses (shortened as S1 to S9, Table S3), and the distribution of these metabolites grouped in each sub class was shown in Table 2.

**Figure 5:**
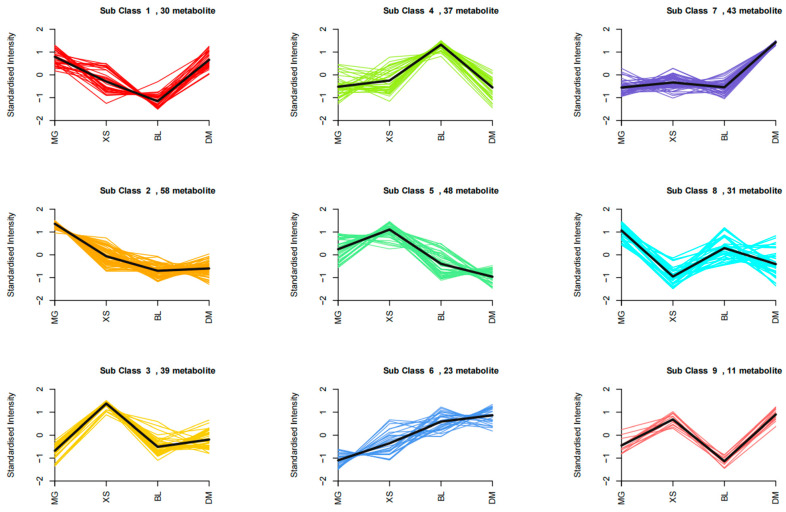
K-means clustering of the metabolites between four pineapple varieties. Nine classes (Sub class 1–9) of 320 metabolites were grouped based on the dynamic changes of metabolites between four pineapple varieties. Colored lines represent the standardized expression patterns of individual metabolites, while the black line indicates the mean trend of each subclass.

DAMs in each sub cluster exhibited similar relative accumulation patterns across the four pineapple varieties (Figure 5). Amino acids and derivatives were primarily enriched in the S5 and S2 subclasses, exhibiting higher abundance in the XS and MG varieties. Phenolic acids were predominantly found in the S2, S3, and S5 subclasses, which also demonstrated greater accumulation in MG and XS. Flavonoids were mainly present in the S8 and S4 subclasses, with both subclasses accumulating more in the BL variety. Alkaloids were more abundant in the S7, S4, and S2 subclasses, exhibiting the highest accumulation in DM, BL, and MG, respectively. The less diverse classes of lignans and coumarins, as well as saccharides and alcohols, were primarily found in the S2 and S3 subclasses, with greater accumulation in MG and XS. These results indicate that the categories and quantities of predominantly accumulated metabolites vary significantly among different varieties, contributing to the formation of their unique qualities.

## 3. Discussion

Pineapple, as a popular tropical fruit crop, is valued for its rich nutritional content, strong aroma, juicy and tender flesh, and balanced sweet-sour taste [3,14]. The introduction of Tainong varieties has become a key measure in recent years to enhance the market competitiveness of mainland varieties in China [4,5,6]. Although various studies have explored the metabolomic changes during pineapple fruit development [14,15], significant differences still persist in the metabolic characteristics between varieties. Identifying specific metabolic profiles not only provides insight into varietal advantages but also offers scientific evidence for market promotion and sales [9]. In this study, we conducted a comparative metabolomic analysis on three promoted pineapple varieties (MG, XS, DM) introduced from Taiwan using the main cultivar (BL) as a control, to elucidate their distinct metabolic features. This analysis aimed to provide a reference for promoting new varieties and guiding consumer preferences.

A total of 551 metabolites were identified across the four varieties, including 100 phenolic acids, 89 amino acids and derivatives, 72 flavonoids, 70 organic acids, 63 alkaloids, 49 saccharides and alcohols, 45 nucleotides and derivatives, 22 lignans and coumarins, five terpenes, two tannins, one quinone, and 33 other metabolites (Table S1, Figure 1). This number of identified metabolites is notably higher than that reported in previous studies on pineapple fruit. For instance, Muhammad Ikram et al. (2020) detected only 47 metabolites in pineapple flesh using a GC-MS approach involving methoximation and MSTFA silylation derivatization [11]. This method is inherently limited to volatile and thermally stable small molecules, thus excluding many non-volatile or large plant secondary metabolites. In contrast, our use of a widely targeted metabolomic approach enabled the detection of a much broader spectrum of compounds, including a substantial number of secondary metabolites. Even when compared with studies employing similar UPLC-MS/MS-based platforms, our dataset remains relatively comprehensive. For example, Hong et al. (2021) identified 466 metabolites in the flesh of the “Comte de Paris” (BL) pineapple during ripening stages, using the same analytical platform provided by Wuhan Metware Biotechnology Co., Ltd. [12]. As metabolomics databases have been continuously updated and expanded, especially with more reference standards for secondary metabolites, the detection coverage has significantly improved over time. This likely contributed to our ability to detect more compounds, including those not previously reported. Additionally, while amino acids and their derivatives were the most abundant category in the study by Hong et al. [12], our results showed that phenolic acids were the most abundant, followed by amino acids and derivatives, flavonoids, and organic acids. This difference may reflect both varietal differences and the inclusion of multiple pineapple cultivars in our study, which likely enhanced the diversity of detectable metabolites.

The metabolite accumulation patterns of different pineapple varieties exhibited significant differences (Figure 2 and Figure 5). Among these, sugars and organic acids played crucial roles in fruit flavor formation, serving as the primary sources of sweetness and acidity in fleshy fruits [25,26]. Ikram et al. (2021) reported significant differences in taste-related metabolites like organic acids, sugars, and amino acids among three pineapple cultivars: Red Spanish, Smooth Cayenne, and Queen [11]. In our study, pairwise comparisons of the four varieties revealed that more than half of the metabolites in the saccharides and alcohols (34, 69.4%) and organic acids (48, 68.6%) categories exhibited no significant variations across the six comparison groups. High-performance liquid chromatography (HPLC) analysis of mature fruit from 26 pineapple varieties showed that sucrose was the major sugar, followed by glucose and fructose, while citric acid was the predominant organic acid, with malic and quinic acids also present [24]. We found no significant differences in these major sugars and organic acids among the four varieties grown under similar cultivation conditions in summer. Similar findings were reported in a quality comparison study between the BL and MD-2 varieties, indicating that despite notable taste differences, their total soluble solids (TSS) and total acidity (TA) at maturity did not differ significantly [14]. These results suggest that under favorable climatic conditions, different pineapple varieties could achieve balanced sugar–acid accumulation in summer.

Amino acids play a dual role in fruit quality, contributing to both nutritional value and the synthesis of plant aroma volatiles [27,28]. Key amino acids involved in the production of these volatiles include essential amino acids such as branched-chain amino acids (L-leucine, L-isoleucine, and L-valine), L-methionine, and aromatic amino acids, notably L-phenylalanine, alongside L-tryptophan and L-tyrosine [27]. Many metabolites in the amino acids and derivatives category (54 out of 89) exhibited significant differences among the four varieties (Table S1). K-means clustering analysis showed that amino acids and derivatives were most abundant in the S5 and S2 subclasses, with higher accumulation in XS and MG (Figure 5). Among the 54 DAMs in amino acids and derivatives category, 21 were most abundant in XS, including L-glutamine, L-lysine, L-arginine, L-phenylalanine, and L-tryptophan, while 18 out of the 54 DAMs in this category were most abundant in the MG variety, including L-valine, L-leucine, L-isoleucine, L-proline, and L-homocysteine (Table S1). The different amino acids that are abundantly accumulated in MG and XS might serve as precursors for various aroma volatiles, potentially contributing to the distinct fragrances of these two varieties.

In addition to primary metabolites, secondary metabolites exhibit considerable diversity in fruits and significantly influence fruit qualities such as antioxidant capacity, color, and flavor [22,24]. We found that, whether comparing the three introduced varieties to BL or comparing the introduced varieties to each other, the identified DAMs primarily belonged to several categories with high metabolite diversity, including phenolic acids, flavonoids, amino acids and their derivatives, and alkaloids (Figure 4). Consistent with findings reported in other plant species, flavonoids and phenolic acids emerged as the most diverse and differentially accumulated metabolite classes among the varieties [8]. These compounds are well known for their important contributions to the sensory attributes and nutritional value of fruits [29]. Flavonoids are particularly noted for their potent antioxidant activities and roles in pigmentation and taste [30], while phenolic acids exhibit antioxidant, antimicrobial, and anti-inflammatory properties [31]. Our comparative analysis revealed that the categories and numbers of advantageously accumulated metabolites varied significantly among the different varieties (Figure 4 and Figure 5, Table S1). Compared with the other three varieties, the MG variety exhibited the highest number of more-accumulated flavonoid metabolites. In addition, except for XS, MG also showed a greater number of more-accumulated phenolic acid metabolites than both BL and DM. The elevated accumulation of these phenolic compounds may contribute to the strong antioxidant potential, deeper coloration, and enhanced flavor complexity of MG, which are traits commonly preferred by consumers. In addition to phenolic compounds, alkaloids represented another important class of secondary metabolites showing variety-specific accumulation patterns. Compared with BL, the introduced varieties DM and XS exhibited a greater number of more-accumulated alkaloid metabolites (Figure 4). Alkaloids are known to influence fruit flavor and many possess bioactive properties related to stress adaptation and human health benefits [32]. Beyond these major categories, a number of DAMs were also identified in the lignans and coumarins category, which included a relatively small total number of metabolites (only 22 in total). All the DAMs in this category were found to accumulate most abundantly in XS or MG (Table S1). Coumarins are known to possess anti-inflammatory and antimicrobial properties, which may improve both the health value and storability of fruits [33]. Lignans, on the other hand, are phytoestrogens with antioxidant activities, further enhancing the functional food potential of these varieties [34]. Taken together, the differential accumulation of these secondary metabolites likely contributes to the distinct coloration, flavor profiles, nutritional quality, and adaptive traits observed in each pineapple variety.

Compared to the main cultivar BL, the three introduced varieties each demonstrated distinct advantages in mainland conditions [4,5,6]. Compared to the main cultivar BL, 174, 167, and 167 DAMs were identified in the introduced varieties MG, XS, and DM, respectively (Figure 4A–C). Notably, the number of more-accumulated DAMs consistently exceeded the number of less-accumulated DAMs in the three introduced varieties compared with BL. The increased accumulation of various metabolites from different categories might contribute to the formation of diversified qualities and flavors in three introduced varieties. However, the main cultivar BL also showed some unique benefits. BL exhibited the highest accumulation of L-ascorbic acid (vitamin C) (Figure 4, Table S1) and relatively abundant levels of many flavonoids (Figure 5, Table S1), which are known for their strong free radical scavenging activities and potential health benefits [23,35]. Thus, the elevated levels of antioxidant metabolites in BL suggest that it still possesses nutritional advantages and market potential as a functional fruit. Each variety exhibited uniquely accumulated metabolites that were potentially associated with its distinct quality traits. For example, jasmonic acid, which plays a key role in plant adaptation to stress conditions [36], was more highly accumulated in DM compared to the other three varieties (Table S1). Among the four varieties, DM was more suitable for production in autumn and winter, with off-season fruits still maintaining good quality. The higher accumulation of jasmonic acid might contribute to its strong adaptability to low temperatures during autumn and winter production. Overall, the distinct metabolite accumulation patterns among varieties help define their unique combinations of sensory, nutritional, and adaptive traits. These findings provide valuable insights into fruit quality formation and offer useful guidance for variety selection in breeding programs and market-oriented production strategies.

## 4. Materials and Methods

### 4.1. Plant Materials

The experimental materials used in this study include four representative pineapple varieties: the main cultivated variety in China, “Comte de Paris” (BL), and three promoted varieties introduced from Taiwan including “Tainong No. 11” (XS), “Tainong No. 23” (MG), and “Tainong No. 13” (DM). All the plants were cultivated at Jinguang Farm in Tanluo Town, Xixiangtang District, Nanning City, Guangxi Province (22.81° N, 107.80° E), a typical pineapple-growing area characterized by a subtropical humid monsoon climate. The site has an average annual temperature of 21.8 °C, relative humidity of 80%, and annual precipitation of 1300 mm. The orchard is located on flat red soil with a pH of 4.5–5.5 and managed using a uniform water and fertilizer integration drip irrigation system. In-season maturing fruits were collected within one week of the commercial harvest period for each variety. Fruit pulp tissue was sampled between the fruit stalk and peel, with three biological replicates prepared for each variety. Samples were rapidly frozen in liquid nitrogen and stored in a −80 °C ultra-low-temperature freezer for later use.

### 4.2. Measurements of Physicochemical Indices

The total soluble solids content was determined directly using a handheld digital refractometer (PAL-1, ATAGO Co., Ltd., Tokyo, Japan). Titratable acidity was measured by titration with sodium hydroxide solution and expressed as a percentage of citric acid equivalents [37]. The average single fruit weight was measured using an electronic balance (JM-B30002, Yuyao Jiming Weighing Calibration Equipment Co., Ltd., Zhejiang, China). Fruit diameter and length were measured using a digital caliper (300 mm, Xifeng Digital Vernier Caliper, Chengdu, China).

### 4.3. Metabolite Extraction and Detection

For each variety, three independent samples of fruit pulp were subjected to freeze-drying and subsequently crushed using a mixer mill (MM 400, Retsch, Haan, Germany) with a zirconia bead for 1.5 min at 30 Hz. A 100 mg portion of the resulting powder was weighed and subjected to metabolite extraction overnight at 4 °C using 1.2 mL of 70% aqueous methanol. After centrifugation at 12,000 rpm for 10 min, the extracts were filtered (SCAA-104, 0.22 μm pore size) before being subjected to UPLC-MS/MS analysis. The pineapple sample extracts were analyzed utilizing a UPLC-ESI-MS/MS system (UPLC, Nexera X2, SHIMADZU Corporation, Kyoto, Japan; MS, QTRAP® 6500+, Applied Biosystems, Foster City, CA, USA). Subsequently, LIT and triple quadrupole (QQQ) scans were conducted on a triple quadrupole-linear ion trap mass spectrometer (Q TRAP, AB Sciex 6500+, Foster City, CA, USA) with an ESI Turbo Ion-Spray interface, operating in positive and negative ion modes, and controlled by Analyst 1.6.3 software (AB Sciex). The chromatographic separation was performed using an Agilent SB-C18 column (1.8 μm, 2.1 mm × 100 mm). The mobile phase consisted of solvent A (0.1% formic acid in water) and solvent B (0.1% formic acid in acetonitrile). The gradient elution program was as follows: 0 min, 5% B; 0–9 min, 5–95% B; 9–10 min, 95% B; 10.1 min, 5% B; 10.1–13 min, 5% B for column re-equilibration. The flow rate was 0.35 mL/min, the injection volume was 2 μL, and the column temperature was maintained at 40 °C. Ion source parameters were set as follows: source temperature 550 °C, ion spray voltage 5500 V (positive)/−4500 V (negative), curtain gas (CUR) 25 psi, gas 1 (GS1) 50 psi, gas 2 (GS2) 60 psi, and collision gas (CAD) set to high. The mass scan mode was MRM (Multiple Reaction Monitoring) [38,39]. Metabolite extraction and identification in pineapple fruit pulp samples were performed by Wuhan Metware Biotechnology Co., Ltd., Wuhan, China, and the metabolites were qualitatively analyzed using their self-built MetWare database.

### 4.4. Metabolomic Data Analysis

Principal Component Analysis (PCA) was conducted using the prcomp function in R (www.r-project.org). Prior to performing PCA, the data were scaled to unit variance. Hierarchical Cluster Analysis (HCA) for both samples and metabolites were executed using the pheatmap package in R [40] and depicted in heatmaps with dendrograms. For HCA, the normalized signal intensities of metabolites (after unit variance scaling) were visualized on a color spectrum. Differentially accumulated metabolites (DAMs) were defined as metabolites with a Variable Importance in Projection (VIP) value ≥ 1 and an absolute Log2 fold change (Log2FC) ≥ 1 in pairwise comparisons between different varieties [38,39]. VIP values were obtained from the Orthogonal Partial Least Squares Discriminant Analysis (OPLS-DA) results, which also included score plots and permutation tests. OPLS-DA analysis was carried out using the R package MetaboAnalystR [41], with data being log2-transformed and mean-centered prior to analysis. To minimize overfitting, a permutation test with 200 iterations was conducted. Comparison and visualization of the DAMs identified in different comparison groups were shown in bar plot generated using ggplot2 package [42] based on R base v4.2.0. To investigate the trends in the relative abundance of changes in metabolites across different samples, the relative abundances of differential metabolites were standardized and centered, followed by K-means clustering analysis. Identified metabolites were annotated against the KEGG Compound Database (http://www.kegg.jp/kegg/compound/; accessed on 31 May 2024). The annotated metabolites were subsequently mapped to the KEGG Pathway Database (http://www.kegg.jp/kegg/pathway.html; accessed on 31 May 2024). Metabolic pathways containing significantly differentially accumulated metabolites were then subjected to Metabolite Set Enrichment Analysis (MSEA), with significance determined using *p*-values derived from hypergeometric tests.

## 5. Conclusions

Different pineapple varieties exhibited distinct flavors and nutritional values, catering to the diverse demands of the market. Based on the results of the comparative metabolomic analysis, 156, 156, and 144 differentially accumulated metabolites (DAMs) were identified in MG, XS, and DM, respectively, compared to the main cultivar BL, and in all three cases, the number of DAMs with higher accumulation exceeded those with lower accumulation. This indicates a clear metabolic enhancement in the introduced varieties. Pairwise comparison results showed no significant differences in the accumulation of key primary metabolites such as sucrose, glucose, fructose, citric acid, and malic acid across the four varieties, suggesting that in-season fruits of all varieties reached a similarly balanced sugar–acid profile under the tested cultivation conditions. The DAMs in different comparison groups all primarily belonged to several major subclasses, including phenolic acids, flavonoids, amino acids and derivatives, and alkaloids, but the preferentially accumulated metabolites in each variety varied greatly. Branched-chain amino acids (L-leucine, L-isoleucine, and L-valine) and many DAMs in categories including flavonoids, phenolic acids, lignans, and coumarins accumulated most abundantly in MG, contributing to the distinct and enriched flavor and nutritional value of this excellent variety. Aromatic amino acids (L-phenylalanine, L-tryptophan) and many DAMs in the phenolic acid, lignans, and coumarins categories accumulated highly in XS, which may be related to its unique flavor profile. In DM, the dominant accumulation of jasmonic acid might contribute to its stronger adaptability to low temperatures during autumn and winter, potentially allowing off-season fruits to maintain good quality. Although the introduced varieties showed metabolite enrichment in multiple categories, the main cultivar BL exhibited the highest accumulation of L-ascorbic acid and relatively abundant flavonoids, reaffirming its strength in antioxidant quality. These findings could provide valuable insights for developing promotion strategies for different varieties and advancing metabolome-based pineapple improvement programs.

## Figures and Tables

**Table 1:** Physicochemical and Morphological Characteristics of Fruits from Four Pineapple Varieties.

Cultivar	Soluble Solids Content (%)	Titratable Acid Content (%)	Average Fruit Weight (g)	Diameter (mm)	Length (mm)
BL	17.8 ± 1.8	0.61 ± 0.07	1043.00 ± 94.92	99.33 ± 4.62	136.25 ± 8.92
MG	17.9 ± 1.4	0.56 ± 0.06	1002.00 ± 165.52	110.46 ± 4.62 **	113.72 ± 9.79 **
XS	17.2 ± 1.8	0.60 ± 0.07	1015.35 ± 137.14	98.90 ± 5.50	132.98 ± 11.29
DM	18.1 ± 1.2	0.53 ± 0.12	1409.04 ± 145.58 **	111.51 ± 4.24 **	151.61±19.54 *

Note: Parameters of four pineapple varieties are shown as mean ± standard deviation (n = 10). Statistical differences were tested relative to the “Comte de Paris” (BL) variety. * indicates significant difference (*p* < 0.05), ** indicates highly significant difference (*p* < 0.01).

**Table 2:** Distribution of the 320 metabolites grouped in different k-means sub classes.

Class	S1	S2	S3	S4	S5	S6	S7	S8	S9	Total
Saccharides and alcohols	0	4	4	1	1	0	2	3	0	15
Organic acids	0	2	3	3	1	7	1	5	0	22
Amino acids and derivatives	6	9	6	2	16	0	10	2	3	54
Nucleotides and derivatives	0	3	5	3	3	3	2	1	2	22
Phenolic acids	7	19	14	7	10	5	8	4	2	76
Flavonoids	8	9	0	10	6	2	4	12	0	51
Alkaloids	3	8	1	8	6	0	10	2	1	39
Lignans and coumarins	2	4	4	0	3	3	1	0	1	18
Terpenoids	1	0	0	0	0	1	2	0	0	4
Quinones	0	0	0	0	0	1	0	0	0	1
Tannins	1	0	0	0	0	0	0	0	0	1
Others	2	0	2	3	2	1	3	2	2	17
	30	58	39	37	48	23	43	31	11	320

## Data Availability

The datasets supporting the conclusions of this article are included within the article and its additional files. The raw metabolomics data generated in this study have been deposited in the China National GeneBank DataBase (CNGBdb) under the accession number CNP0007659 (https://db.cngb.org/mycngbdb/submissions/project/CNP0007659; accessed on 31 May 2025).

## Supplementary Materials

The following supporting information can be downloaded at: https://www.mdpi.com/article/10.3390/plants14152400/s1, Figure S1: OPLS-DA analyses of the comparison groups between three introduced varieties; Table S1: The identified metabolites in four pineapple varieties and the differentially accumulated metabolites (DAMs) across all six comparative groups; Table S2: The KEGG enrichment analysis of differentially accumulated metabolites (DAMs) identified between different varieties; Table S3: K-means clustering of the metabolites between four pineapple varieties.
