# Supplementary figures and images for: Comparative Metabolomics Analysis of Four Pineapple (Ananas comosus L. Merr) Varieties with Different Fruit Quality
Source: Plants (Basel). 2025 Aug 3;14(15):2400. doi: 10.3390/plants14152400 (PMC12349519; doi:10.3390/plants14152400)

H

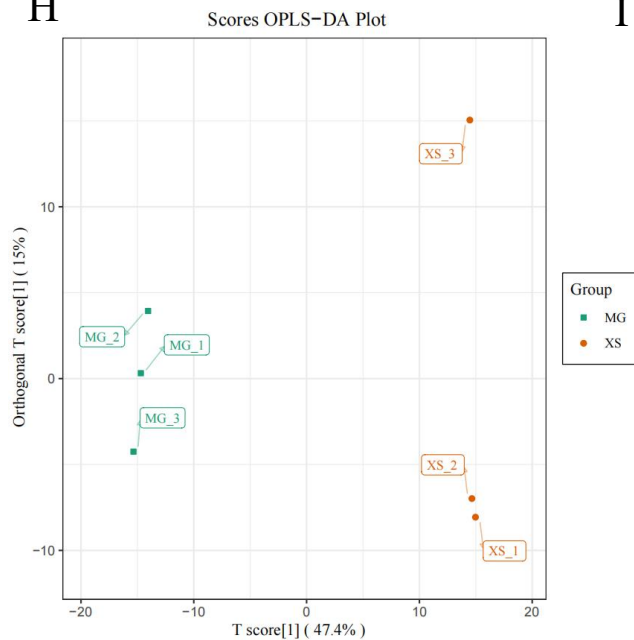

I

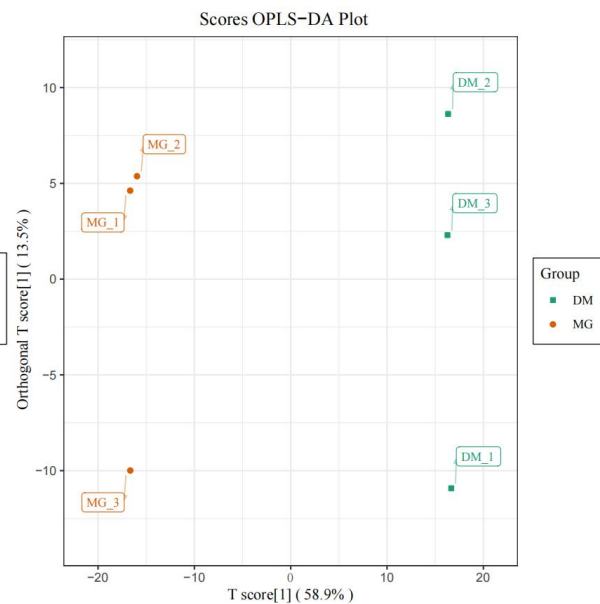

J

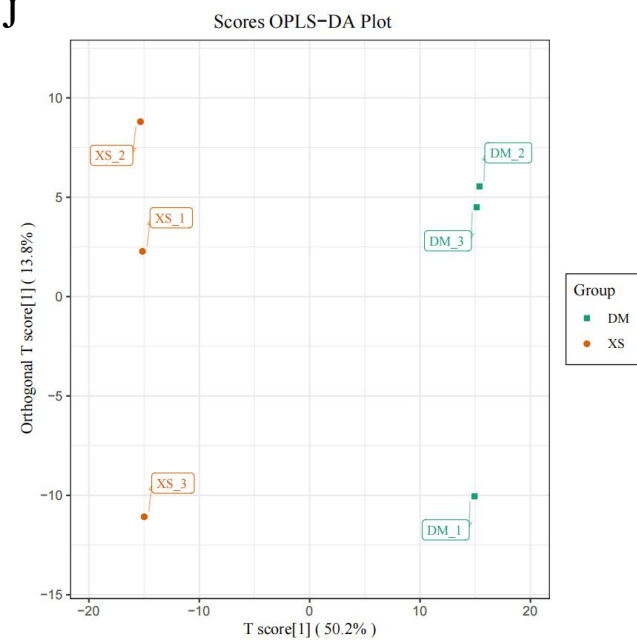

K

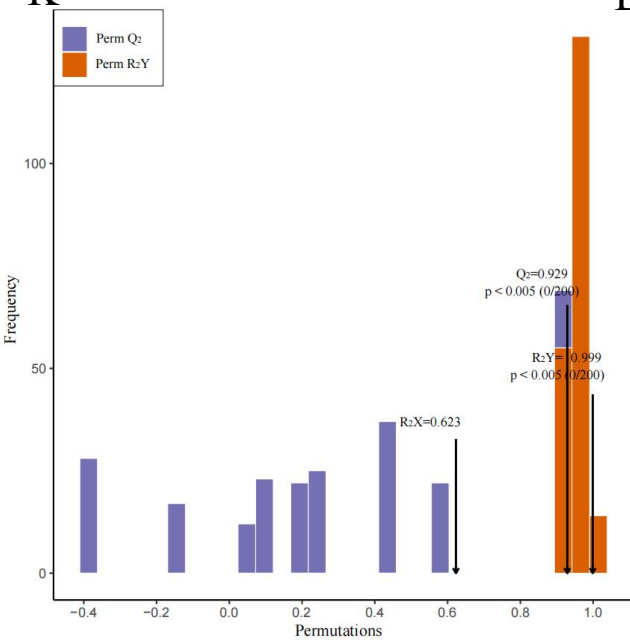

L

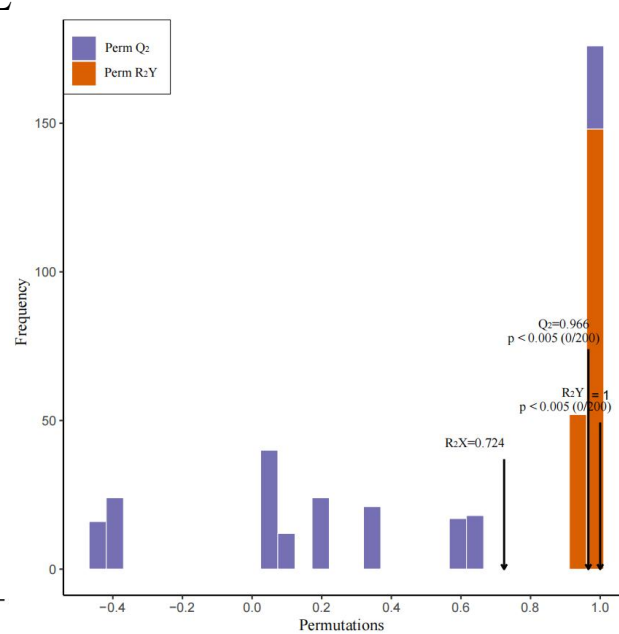

M

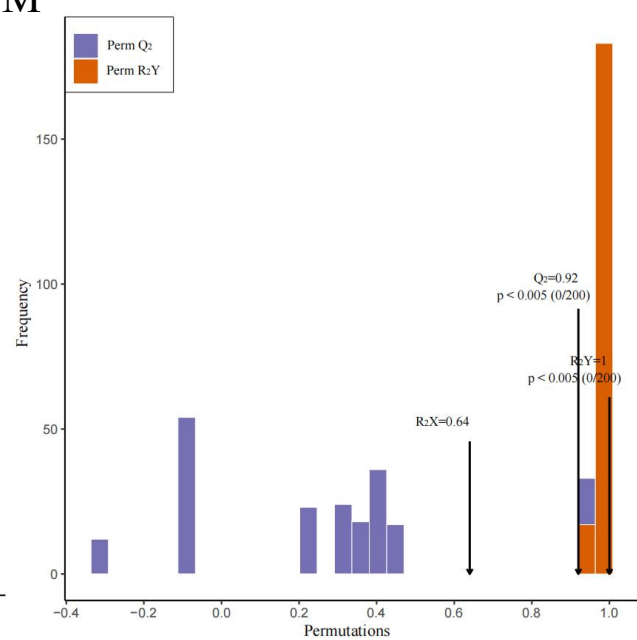

Supplement: Supplementary file 1 [file plants-14-02400-s001.zip › Figure S1.pdf]
